# Impact of Fermentation on the Recovery of Antioxidant Bioactive Compounds from Sea Bass Byproducts

**DOI:** 10.3390/antiox9030239

**Published:** 2020-03-15

**Authors:** Francisco J. Martí-Quijal, Adrián Tornos, Andrea Príncep, Carlos Luz, Giuseppe Meca, Paola Tedeschi, María-José Ruiz, Francisco J. Barba

**Affiliations:** 1 Nutrition, Food Science and Toxicology Department, Faculty of Pharmacy, Universitat de València, Avda. Vicent Andrés Estellés, s/n, 46100, Burjassot, València, Spain; francisco.j.marti@uv.es (F.J.M.-Q.); tornos@alumni.uv.es (A.T.); anprince@alumni.uv.es (A.P.); carlos.luz@uv.es (C.L.); giuseppe.meca@uv.es (G.M.); m.j.ruiz@uv.es (M.-J.R.); 2 Department of Chemistry and Pharmaceutical Sciences, University of Ferrara, 35 - 44121 Ferrara, Italy; tdspla@unife.it

**Keywords:** fermentation, fishing industry byproducts, bioactive peptides, antioxidant activity, phenolic acids

## Abstract

The aim of the present research was to obtain antioxidant compounds through the fermentation of fish byproducts by bacteria isolated from sea bass viscera. To that purpose, bacteria from sea bass stomach, intestine, and colon were isolated. With the selected bacteria, growing research was undertaken, fermenting different broths prepared with sea bass meat or byproducts. After the fermentation, the antioxidant activity, phenolic acids, and some proteins were evaluated. The main phenolic acids obtained were DL-3-phenyl-lactic acid and benzoic acid at a maximum concentration of 466 and 314 ppb, respectively. The best antioxidant activity was found in the extracts obtained after the fermentation of fish byproducts broth by bacteria isolated from the colon (6502 μM TE) and stomach (4797 μM TE). Moreover, a positive correlation was found between phenolic acids obtained after the fermentation process and the antioxidant activity of the samples. It was also concluded that the lactic acid bacteria isolated from sea bass had an important proteolytic capacity and were able to synthesize phenolic acids with antioxidant capacity. This work has shown the relevance of fermentation as a useful tool to valorize fish byproducts, giving them an added economic value and reducing their environmental impact.

## 1. Introduction

The latest FAO (Food and Agricultural Organization) report, published in 2018, showed an important growth in fish caught over the last few years due to the increased fish population’s consumption (from 17.6 kg per capita in 2007 to 20.3 kg per capita in 2016) [1].

During the fish production process, several byproducts are produced, representing more than 30% of fish weight, and in some cases, up to 70% [2]. Among the different parts of fish that can be considered byproducts, muscle pieces, viscera, thorns, heads, skin, fins, and scales are the most representative [3]. These must be properly managed in order to avoid environmental problems and to maintain the sustainability of the resources. Moreover, it should be noted that since each by-product has a different composition, different alternatives have been evaluated for each of them [4].

For instance, different innovative approaches have been studied in order to valorize fish byproducts due to the environmental problem they represent; for instance, the application of green extraction technologies such as ultrasound-assisted extraction, pulsed electric fields, and supercritical fluid extraction to obtain valuable compounds from fish byproducts. These technologies allow the recovery of interesting compounds with the minimum environmental impact, reducing the use of organic solvents, which can be toxic and/or improving the extraction efficiency [5,6].

Fermentation is conventionally used for food preservation, but not so frequently for valorization purposes, as a tool to obtain high added-value compounds. In the food industry, fermentation is considered as any process in which the activity of microorganisms promotes the development of a profitable change in a food or drink [7]. Through fish byproducts, fermentation is possible to obtain quite different high added-value compounds such as bioactive peptides, high-quality oils, or protein hydrolysates, as well as many others like bioplastics, lactic acid, or preservative compounds, which are very useful in food, pharmaceutical or cosmetic industries [8]. For fermentation processes, the main microorganisms used are lactic acid bacteria (LAB), mainly due to their safety, since they are present in a multitude of fermentative processes of food aimed for human consumption, such as dairy products or alcoholic beverages.

The LAB is a group of microorganisms characterized by the production of lactic acid as the main product of carbohydrate fermentation. They are Gram-positive, not sporulated, and have a coralline or bacillary shape [9,10,11]. These bacteria, through the phosphorylation of carbohydrates, obtain the metabolic energy forming the main metabolite, lactic acid. As the acid is accumulated, the structure of the proteins is modified, as happens with the texture of the product. Such bacteria have been used throughout history for the fermentation of a multitude of foods, producing changes in taste and texture, acting as a preservative, and increasing their shelf life [9,12].

Among the different high added-value compounds obtained from fish byproducts, it is possible to highlight nutrients (e.g., proteins and lipids) as well as bioactive compounds (e.g., polyphenols and bioactive peptides). For instance, during fermentation, LAB play a very important role due to their proteolytic capacity, which allows them to fractionate the proteins into peptides and free amino acids. Generally, small peptides of 3 to 20 amino acids are obtained [13,14]. Some of these peptides have biological activity; therefore, they are considered bioactive peptides [15]. Furthermore, not only are bioactive peptides produced during fermentation, but different phenolic acids have also been found, such as benzoic acid or phenyl-lactic acid, identified in silages inoculated with LAB [16]. These compounds produced by the LAB are also related to the antimicrobial activity and the antioxidant activity [17].

Sea bass is one of the most produced fish worldwide, but especially in Europe. The main production system is aquaculture, with a production of 165,915 tons in 2016, representing 96% of the total production [18]. Sea bass culture is mainly focused on the geographical area of the Mediterranean sea, with Turkey, Egypt, Tunisia, Italy, and Spain being the main producers [19]. Due to the new trends in both consumption and commercialization (for example, the sale of frozen fillets), a large number of byproducts are generated during the sea bass production process, which needs to be managed properly, assuming an additional cost for the processing industries [20]. In fact, studies have already been carried out focusing on obtaining high added-value compounds from seabass byproducts [20,21].

Therefore, the present study aims to explore the potential application of fermentation, using LAB isolated from sea bass viscera, in order to valorize sea bass byproducts by obtaining high added-value antioxidant compounds.

## 2. Materials and Methods

### 2.1. Samples

Sea bass samples were purchased from a local supermarket, and they were obtained from the Mediterranean Sea. They were quickly preserved using an ice bath after their death. The same day the samples were obtained, they were eviscerated, and the meat was separated from the byproducts (skin, head, tail, thorns, and backbone). Subsequently, all the parts, except the viscera, were frozen until needed for use.

### 2.2. Chemicals

The radical ABTS (2,2′-azino-bis (3-ethylbenzthiazoline-6-sulfonic acid)), Trolox (6-hydroxy-2,5,7,8-tetramethylchroman-2-carboxylic acid), Folin-Ciocalteau reagent 1N, gallic acid, and potassium persulfate (K_2_S_2_O_8_) were purchased from Sigma–Aldrich (Steinheim, Germany). The Gram color kit containing crystal violet, Lugol PVP, safranin, and decolorizing solution was purchased from Liofilchem Bacteriology Products (Roseto, Italy). Anaerocult^®^ anaerobic environment system was purchased from Merck (Darmstadt, Germany). The enzyme ammonium persulfate (APS) and tetramethylethylenediamine (TEMED) catalyst used for the polymerization of polyacrylamide gels were obtained from Sigma-Aldrich (St. Louis, USA). Acetonitrile and dithiothreitol (DTT) were purchased from VWR (Leuven, Belgium). The molecular weight pattern 10−250 kDa was obtained from BioRad (Hercules, California). Ethanol 96° was purchased from GUINAMA S. L. U. (Valencia, Spain).

### 2.3. Isolation of Bacteria

Once the fish samples were eviscerated, those bacteria existing in the digestive tract were isolated. For that purpose, the digestive system of the sea bass samples was separated into three parts, the stomach, small intestine, and colon. Each of these parts was immersed in 200 mL of Man, Rogosa, and Sharpe broth (MRS Broth, Liofilchem Bacteriology Products, Roseto, Italy) and incubated at 37 °C for 48 h under anaerobic conditions, using an Anaerocult^®^ device. Then, serial dilutions were made and growth in Petri dishes with Man, Rogosa, and Sharpe agar (MRS Agar, Liofilchem Bacteriology Products, Roseto, Italy) and incubated at 37 °C for 48 h. Finally, 8 strains were isolated from each part of the digestive tract, and they were identified using a code about the part of the intestinal tract from where they were isolated (“S” for stomach, “I” for small intestine, and “C” for colon) followed each one by a number. All these operations were carried out under sterile conditions in a Telstar MH 100 laminar flow hood (Telstar, Terrassa, Spain).

### 2.4. Identification of Bacteria

To identify the type of bacteria isolated, the colonies were characterized by their characteristic morphologies and their type of fermentation [22,23]. For the analysis of morphological characteristics, shape, surface, edge, and color of the colonies were considered. Moreover, a Gram stain was performed to study the characteristics of the cell membrane and the morphology of the bacteria. The production of acid during fermentation was measured by the decrease of pH.

In order to identify bacteria genetically, a 16S rRNA gene sequencing was performed. The identification of isolates was performed using the method described by Chenoll et al. [24] with some modifications. Briefly, the main modification was the primers; in the cited reference, the primers used were 616V and 630R, however, in our case, we used 616V and 699R. The DNA culture was extracted using the high pure PCR template preparation kit (Roche). The 16S rRNA sequence was amplified and sequenced using an Applied Biosystems ABI PRISM BigDye terminator cycle sequencing ready reaction kit (Foster City, CA, USA). The DNA templates were amplified by PCR using the universal primers amplifying a 1000 bp region of the 16S rRNA gene; 616V: 5′-AGAGTTTGATYMTGGCTCAG-3′ for the forward primer and 699R: 5′-RGGGTTGCGCTCGTT-3′for the reverse primer. The 616V and 699R primers, Taq DNA polymerase, and dNTP mix were obtained from Thermo Fisher Scientific (Waltham, MA, USA). The DNA templates were amplified by initial denaturation at 94 °C for 10 min, followed by 40 cycles of denaturation at 94 °C for 1 min, annealing at 55 °C for 1 min, extension at 72 °C for 1 min and a final extension at 72 °C for 10 min. The integrity of the PCR products was assayed by the development of single bands following electrophoresis for 1 h at 100 V in 2% (w/v) agarose gels in Tris–borate EDTA buffer. Amplicons were purified using the commercial Metabion GmbH mi-PCR purification kit (Planegg, Germany), followed by sequencing reactions using the BigDye terminator v3.1 cycle sequencing kit (Applied Biosystems), premixed format. The resulting sequences were automatically aligned and inspected visually and then compared with the on-line tool BLAST [25]. The strain was identified based on the highest scores. The isolated and identified bacteria are kept at the Department of Preventive Medicine and Public Health (Faculty of Pharmacy, Universitat de València) and are available to any research group which is interested in working with them.

### 2.5. Elaboration of Fish Meat and Fish Byproducts Broths

Four different growth broths were elaborated: waste broth (WB; fish byproducts), meat broth (MB; fish fillets), waste broth 2% glucose (WBG; fish byproducts + 2% glucose), and meat broth 2% glucose (MBG; fish fillets + 2% glucose).

The procedure carried out to prepare the broths was the same in all of them. Firstly, an Oster 4655 crusher (London, United Kingdom) was used for crushing fish byproducts or meat, following a ratio of 1:3 (w/v) with water. Subsequently, the broths were centrifuged (4000 rpm, 15 min) using an Eppendorf 5810R centrifuge (Hamburg, Germany). The supernatants were poured into 1 L bottles, and 2% glucose was added to the corresponding broths. Then, they were pasteurized using an SBS30 bath (Staffordshire, United Kingdom) at 85 °C for 20 min [26]. Once pasteurized, they were again centrifuged at 4000 rpm for 15 min. The different broths were frozen until use.

### 2.6. Fermentation

To carry out the fermentation process, 2 mL of exponentially growing bacteria (37 °C for 12 h) were inoculated in 100 mL MRS Broth at a final concentration of 10^7^ CFU/mL and incubated in different tubes at 37 °C for 24, 48, and 72 h. To verify that the fermentation was performed appropriately, the pH was measured, and the bacterial load count was carried out every 24 h.

After the incubation period, the tubes were centrifuged in an Eppendorf 5810R centrifuge (Hamburg, Germany) at 4000 rpm for 10 min. The supernatants, once cleaned of cells, were frozen at −20 °C.

### 2.7. SDS-PAGE Electrophoresis

In order to evaluate the different sizes of fish protein residues after 72 h fermentation, SDS-PAGE electrophoresis was performed according to the method previously described [27].

### 2.8. Extraction and Identification of Phenolic Acids

To determine the phenolic acids produced by the LAB, the method described by Denardi-Souza et al. [28] was carried out. For that purpose, LC-ESI-qTOF-MS was used.

The equipment used for the analysis consisted of LC Agilent 1200 (California, USA) chromatography. The column used was a Gemini C18 50 × 2 mm, with 100 Å and a particle size of 3 μm (Phenomenex). The mobile phases used were water as solvent A and acetonitrile as solvent B, both acidified with 0.1% formic acid. The chosen elution gradient was 0 min, 5% B; 30 min 95% B; 35 min, 5% B, at a flow rate of 0.3 mL/min. Finally, the volume injected was 20 μL, and the column equilibrated 3 min before the next analysis.

Mass spectrometry was performed with an Agilent ultra high definition accurate mass Q-TOF-MS 6540, equipped with Agilent dual jet stream (Dual AJS ESI) electrospray ionization source. It was programmed in the negative ionization mode with the following conditions: drying gas flow at 8 L/min, drying gas temperature at 350 °C, nebulizer pressure at 30 psi, capillary voltage at 3.5 kV, voltage fragmentor at 175 V, and scan range from 20 to 380 m/z. Collision energies of 10, 20, and 40 eV were used for the targeted MS/MS experiments. Finally, integration and data elaboration were managed using the Masshunter qualitative analysis B.08.00 software.

### 2.9. Antioxidant Activity

To determine the total antioxidant capacity, the TEAC (Trolox equivalent antioxidant capacity) assay was used as previously described [29].

Briefly, 25 mL of ABTS (7 mM) was mixed with 440 μL of K_2_S_2_O_8_ (140 mM) and kept at room temperature for 12–16 h in darkness to obtain the radical ABTS•^+^. This solution was diluted 1/100 in ethanol in order to obtain a working solution with an absorbance of 0.700 ± 0.020 measured at 734 nm (A_0_, initial absorbance). Then, 100 μL of the appropriately diluted extracts were added, and the absorbance was measured at 3 min (A_f_) in a Perkin–Elmer UV/Vis Lambda 2 spectrophotometer (Perkin–Elmer, Jügesheim, Germany). The measurements were made in triplicate. The percentage of inhibition (% Inhibition) was calculated using the following equation (1);

(1) $$\%\;\mathrm{Inhibition}=\left(1-\frac{A_{f}}{A_{0}}\right)\times 100$$

In order to obtain the antioxidant capacity, a standard curve was first established with Trolox, and the percentage of inhibition of the samples was interpolated. The results were expressed as micromolar Trolox equivalent (μM TE).

### 2.10. Statistical Analysis

The statistical analysis was performed using the InfoStat^®^ software version 2018. All experiments were performed in triplicate, and the differences between the groups were analyzed using a one-way analysis of the variance (ANOVA) followed by the Tukey HSD post-test for multiple comparisons. The level of significance considered as *p* ≤ 0.05.

The correlations were established using the StatAdvisor^®^ software version 2018 and Pearson’s test. The range of correlation coefficients ranged from −1 to +1, and they measured the strength of the linear relationship between the variables. *p* values ≤ 0.05 indicate correlations significantly different from zero, with a confidence level of 95.0%.

## 3. Results and Discussion

### 3.1. Isolation and Identification of Bacteria

Although 64 different strains were initially isolated (8 from each part of the digestive tract of different sea bass fishes), only 30 were finally obtained (7 from the stomach, 7 from the intestine and 16 from the colon), due to the rest having either stopped growing or not showing adequate growth. After the strains were correctly isolated and identified, different aspects of the colonies were analyzed. All had a white, circular shape and were opaque. In addition, they did not present any type of pigment, which fits perfectly with the description of LAB provided by Astuti [22].

Subsequently, a Gram stain of each of the isolated colonies was performed, and, as a result, Gram-positive cocci and bacilli were obtained. After the first identification of the morphology, different bacteria from different parts were selected and fermented with two types of broths to see if the pH was modified. The pH decreased independently of the colonies studied as fermentation time increased, whereas this effect was not observed in the controls (Table 1).

With the results obtained after carrying out the above-mentioned tests, it could be concluded that the isolated bacteria could belong to the LAB family since their morphology was identical to the LAB, the compounds produced during the fermentation were acidic and promoted a decrease in pH, and also because they were isolated in MRS medium, which is suitable for LAB growth.

However, in order to obtain a clear bacteria identification, a 16S rRNA gene sequencing was performed for four of these bacteria. The results obtained showed that isolated bacteria were *Lactobacillus plantarum*, concluding that the isolated bacteria were LAB. For instance, several authors have isolated lactic bacteria (mainly *Streptococcus*, *Enterococcus*, *Lactobacillus*, *Carnobacterium,* and *Lactococcus* genera) from the intestinal tract of different types of fish [11,30,31], and the stomach, intestine, and gills using the same method [32].

### 3.2. Fermentation

Regarding the results of the bacterial load test performed to choose the best culture broth, as can be seen in Table 2, there were no statistically significant differences (*p* > 0.05) between the broths containing 2% glucose (MBG and WBG) and those that do not (MB and WB).

According to Table 2, there were no significant differences between any of the broths tested. However, it was finally decided to use those samples containing 2% glucose in order to provide more carbohydrates to the broth, and thus ensuring bacterial growth.

### 3.3. Electrophoresis

**Figure 1:**
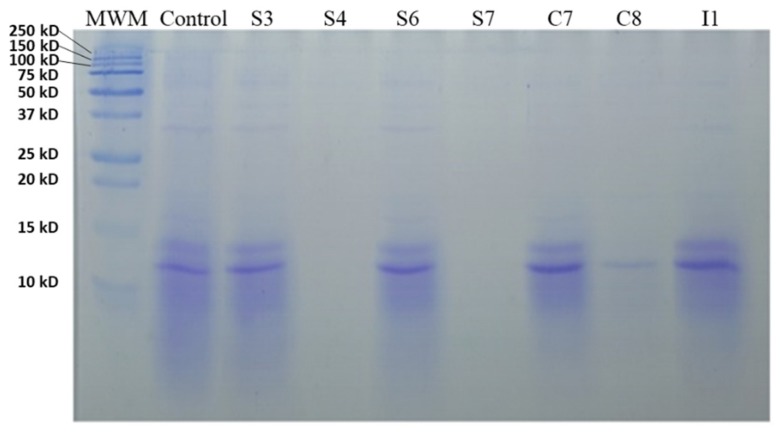
SDS-PAGE analysis of fermented waste broth with 2% glucose with different isolated bacteria; MWM, molecular weight marker.

Regarding the results obtained after using the electrophoresis, a great difference was observed in the protein profile between the different fermented broths. The fermented MBGs had higher protein concentration and molecular weight than the WBG, which showed a reduced protein concentration and lower molecular weight (10−15 kDa). Figure 1 shows the proteolytic activity of different bacteria strains on the WBG. As can be seen in Figure 1, the strains S4, S7, and C8 showed hydrolysis of the proteins present in the WBG. However, the rest of the fermented WBGs did not show great differences compared to the control.

**Figure 2:**
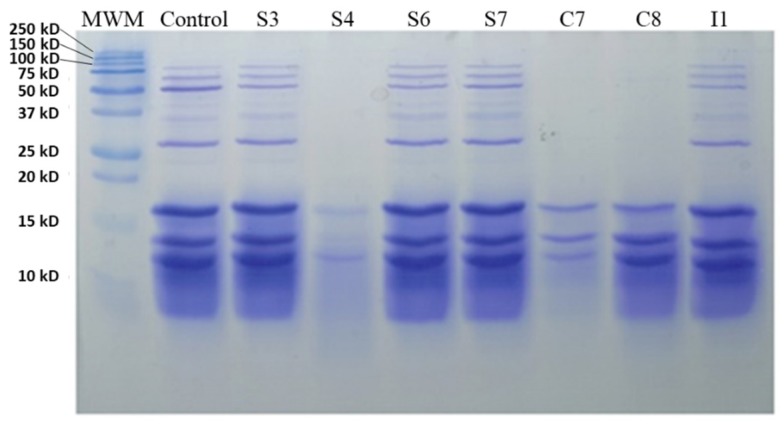
SDS-PAGE analysis of fermented meat broth with 2% glucose with different isolated bacteria; MWM, molecular weight marker.

Regarding the MBG (Figure 2), there was greater proteolysis when strains S4, C7, and C8 were used; thus, promoting the hydrolysis of proteins of high molecular weight (20−250 kDa). The rest of the strains had a similar amount of protein, like the control, despite the fermentation process.

The results obtained in our study were similar to previous works carried out by other authors. For instance, Altinelataman et al. [26] analyzed the protein profile of sea bass and sea bream muscle hydrolysates by SDS-PAGE. These authors concluded that the peptides often appeared to be included within larger fragments because incomplete hydrolysis occurred. In the same study, the authors were able to identify different proteins such as parvalbumin beta-2 (15 kD) or triosephosphate isomerase B (24−26 kD), which is in accordance with our results.

### 3.4. Phenolic Acids

Two phenolic acids, DL-3-phenyllactic acid, and benzoic acid, were isolated from the broths used in this study resulting from the fermentation process. The results showed an increase in the content of these compounds was obtained during the fermentation time, thus, it was demonstrated that these acids were products of fermentation since these compounds were not found in any of the control samples.

As shown in Table 3, the bacteria isolated from the stomach produced the greatest amount of phenolic acids in MBG and WBG, being the predominant compound DL-3-phenyllactic acid, reaching up to 467 ppb when the S7 strain was used. Benzoic acid was also found, with lower values, up to 314 ppb when the S4 strain was used. Both phenolic acids increase when fermentation time increases.

Other authors have studied the production of phenolic acids after using the fermentation process in different matrices. For example, Lavermicocca et al. [33] observed an important amount of DL-3-phenyllactic acid after fermentation with 9 LAB in MRS. On the other hand, the study by Lau and Liong [34] concluded that the concentrations of the acids produced by the LAB species differed according to the strain studied. Urbiene and Leskauskaite [35] investigated the formation of benzoic, sorbic, and nucleic acids during fermentation with LAB. They found concentrations up to 14–23 mg/kg of benzoic acid in the milk fermented by LAB.

In the study conducted by Quattrini et al. [36], the potential of 25 strains of *Lactobacillus plantarum* was explored. They were able to produce 1,2-dihydroxybenzene, benzoic acid, p-hydroxyphenyllactic acid, and DL-3-phenyllactic, which agree with our results. Ramos et al. [37] studied strains of *Lactobacillus plantarum*, finding that these strains produced mevalonolactone, 5-methyl-hydantoin, and benzoic acid. Moreover, Yu et al. [38] examined the use of *Lactobacillus rhamnosus*, *Lactobacillus paracasei*, and *Streptococcus thermophilus* at different incubation temperatures and optimized the production of benzoic acid.

### 3.5. Antioxidant Activity

Table 4 shows the results of the antioxidant activity of the fermented samples. As can be seen, the results are quite different, reaching the highest antioxidant activity after using a fermentation process of 24 h. The only sample that achieved significant differences in antioxidant activity respect to control was C8. The C7 sample, and all those from the stomach (S), reached values with a large difference compared to control after 48 h of fermentation. A similar response was observed for stomach samples (S) after 72 h. Considering the results obtained, it can be affirmed that, in the case of MBG, the samples with the highest antioxidant activity are those that have been fermented by bacteria from the stomach (Table 4).

As for the samples obtained from the WBG (Table 5), the strains S3 and I1 stand out and showed a greater antioxidant activity compared to the control (without bacteria) samples. With this broth, the highest antioxidant activity was reached at 48 h. The C8 strain showed the highest activity of all samples after 72 h of fermentation. Once again, bacteria from the stomach are, in general, the ones with the greatest antioxidant activity.

The results obtained in this study were in close agreement with others previously obtained by different authors such as Sampath et al. [39], who obtained similar results in extracts obtained after the fermentation with LAB of viscera of horse mackerel. In another study, Altinelataman et al. [26] found antioxidants at maximum concentrations, between 0.25 and 0.5 mg/mL, in hydrolyzed sea bass. Sae-Leaw et al. [40] and Vázquez et al. [41] also found similar results with broths prepared with hydrolyzed gelatine and fermented byproducts with LAB of hake, grenadier fish, and horse mackerel among others.

Moreover, Raghavan et al. [42] showed an important antioxidant activity in tilapia hydrolysates. In addition, there are more studies that found antioxidant peptides in the skin of blacktip shark, blue mussel protein, cod protein, *Trichiurus japonicus* protein, skate skin, and oysters [43,44,45].

### 3.6. Correlations between Phenolic Acids and Total Antioxidant Capacity

In order to know the correlations between the different antioxidant compounds studied and the total antioxidant capacity (TAC), Pearson’s test was performed. Significant positive correlations (*p* ≤ 0.05) were found between benzoic acid and TAC (*r* = 0.2967) in fermented MBG. On the other hand, the correlation between DL-3-phenyllactic acid and TAC was also significant (*p* ≤ 0.05), but it showed a negative correlation (*r* = −0.2744).

However, when the existing correlations in phenolic acids and TAC of the fermented WBG were evaluated, the different behavior was evidenced. There was no significant correlation between benzoic acid and TAC (*r* = 0.1099; *p* > 0.05). Nevertheless, there was significant (*p* ≤ 0.0001) and positive correlation between DL-3-phenyllactic acid and TAC (*r* = 0.5793). The difference between the results obtained in both samples may be due to the difference in the composition of the broths.

Although no studies have been found that relate the presence of phenolic acids with antioxidant activity in sea bass byproducts, correlations were observed between phenolic compounds and antioxidant capacity in other marine and vegetable food products (e.g., *Laminaria* and *Porphyra* algae) [17,46,47,48].

## 4. Conclusions

In conclusion, it has been shown that the fermentation of fish byproducts with lactic acid bacteria (LAB) is a useful tool for obtaining antioxidant compounds. As it has been seen, there is a strong positive correlation between DL-3-phenyllactic acid and the total antioxidant capacity. In addition, DL-3-phenyllactic acid is obtained as a result of the fermentation of fish byproducts, which is an economical, clean, and environmentally friendly process. For all these reasons, the fermentation of fish byproducts can be a good strategy for the reduction of fish byproducts through the valorization of them, contributing to achieving sustainable development.

## Figures and Tables

**Table 1:** pH results during fermentation.

Sample	0h	24 h	48 h	72 h
MB CONTROL	6.97	6.98	6.89	6.84
WB CONTROL	6.87	6.80	6.76	6.63
S3 MB	6.97	6.36	6.01	5.71
S3 WB	6.87	6.15	5.92	5.70
S4 MB	6.97	5.70	3.99	3.83
S4 WB	6.87	5.55	3.80	3.73
S6 MB	6.97	6.34	5.93	5.69
S6 WB	6.87	6.27	5.81	5.53
S7 MB	6.97	6.75	4.45	4.73
S7 WB	6.87	6.67	5.10	3.74
I1 MB	6.97	6.60	5.99	5.68
I1 WB	6.87	6.43	5.80	5.55
C7 MB	6.97	5.54	4.45	4.30
C7 WB	6.87	5.45	5.10	5.23
C8 MB	6.97	6.45	6.12	4.54
C8 WB	6.87	6.30	4.38	4.35

MB, meat broth; WB, waste broth; S, bacteria from stomach; I, bacteria from small intestine; C, bacteria from colon.

**Table 2:** Bacterial load (logarithms) from the stomach, intestine, and colon.

Heading	MB	MBG	WB	WBG
**Stomach bacteria**	9.96 ± 1.63 ^A^	10.23 ± 1.62 ^A^	9.97 ± 2.13 ^B^	9.93 ± 1.62 ^B^
**Intestine bacteria**	8.92 ± 3.84 ^A^	7.85 ± 1.69 ^A^	9.21 ± 1.80 ^B^	9.36 ± 2.01 ^B^
**Colon bacteria**	10.35 ± 1.30 ^A^	10.47 ± 2.53 ^A^	10.19 ± 2.69 ^B^	10.84 ± 1.32 ^B^

Different letters in the same column mean statistically significant differences (*p* ≤ 0.05). MB, meat broth; MBG, meat broth with 2% glucose; WB, waste broth; WBG, waste broth with 2% glucose.

**Table 3:** Phenolic acids in fermented and control samples.

Samples		DL-3-phenyllactic Acid			Benzoic Acid		
		24 h	48 h	72 h	24 h	48 h	72 h
**Meat broth (MBG)**	**CONTROL**	nd	nd	nd	nd	nd	nd
	**S3**	11.4 ± 2.5 ^b^	30.9 ± 1.2 ^d^	33.0 ± 1.1 ^b^	52.8 ± 4.3 ^c^	101.0 ± 2.5 ^f^	166.4 ± 2.2 ^e^
	**S4**	nd	12.7 ± 0.4 ^b^	29.4 ± 0.7 ^b^	76.4 ± 3.6 ^d^	179.8 ± 3.4 ^g^	314.2 ± 5.7 ^f^
	**S6**	64.1 ± 1.9 ^d^	109.5 ± 3.5 ^g^	289.9 ± 1.6 ^f^	29.0 ± 4.2 ^b^	19.4 ± 0.6 ^b^	12.9 ± 0.9 ^b^
	**S7**	21.0 ± 1.5 ^c^	192.9 ± 2.3 ^h^	466.7 ± 3.5 ^g^	35.0 ± 0.7 ^b^	51.7 ± 2.3 ^d^	51.0 ± 2.4 ^c^
	**I1**	10.5 ± 1.2 ^b^	20.7 ± 2.2 ^c^	163.0 ± 4.1 ^e^	nd	66.5 ± 2.3 ^e^	13.2 ± 0.8 ^b^
	**C7**	9.6 ± 2.6 ^b^	38.4 ± 2.3 ^e^	76.7 ± 1.4 ^d^	0.7 ± 0.3 ^a^	4.5 ± 0.8 ^a^	4.6 ± 0.2 ^a^
	**C8**	20.8 ± 1.2 ^c^	49.7 ± 2.1 ^f^	51.7 ± 1.1 ^c^	4.7 ± 0.9 ^a^	28.0 ± 2.2 ^c^	92.1 ± 2.3 ^d^
**Waste broth (WBG)**	**CONTROL**	nd	nd	nd	nd	nd	nd
	**S3**	21.9 ± 1.5 ^b^	145.4 ± 4.2 ^d^	138.4 ± 4.2 ^e^	172.7 ± 2.6 ^f^	59.5 ± 4.3 ^c^	74.8 ± 6.7 ^e^
	**S4**	23.1 ± 1.1 ^b,c^	186.4 ± 2.4 ^e^	71.8 ± 2.2 ^c^	117.9 ± 4.3 ^e^	101.8 ± 3.3 ^c^	48.7 ± 1.1 ^d^
	**S6**	26.5 ± 2.7 ^c,d^	22.6 ± 3.6 ^b^	20.1 ± 1.2 ^b^	89.5 ± 4.7 ^c^	82.3 ± 5.2 ^c^	92.2 ± 6.6 ^f^
	**S7**	21.0 ± 1.1 ^b^	19.7 ± 5.4 ^b^	146.7 ± 3.2 ^f^	101.9 ± 2.5 ^d^	7.6 ± 1.6 ^a,b^	17.5 ± 4.4 ^b^
	**I1**	30.7 ± 2.1 ^d^	160.1 ± 1.5 ^d^	175.0 ± 3.3 ^g^	88.9 ± 5.2 ^c^	64.7 ± 3.3 ^c^	21.3 ± 3.1 ^b,c^
	**C7**	53.8 ± 1.3 ^e^	65.8 ± 2.3 ^e,c^	81.5 ± 0.6 ^d^	44.8 ± 2.2 ^b^	22.8 ± 2.1 ^b^	25.4 ± 3.2 ^b,c^
	**C8**	28.0 ± 1.2 ^d^	80.5 ± 3.3 ^c^	198.2 ± 4.5 ^h^	460.5 ± 3.3 ^g^	120.0 ± 5.2 ^d^	32.5 ± 4.1 ^c^

Results expressed in ppb, parts per billion. Different letters in the same column mean statistically significant differences (*p* ≤ 0.05); nd, not detected.

**Table 4:** Antioxidant activity (μM Trolox equivalent) of the meat broth with 2% glucose fermented by the selected bacteria at 37 °C for 24, 48, and 72 h.

Sample	24 h	48 h	72 h
**Control**	793.12 ± 63.54 ^a^	463.70 ± 22.12 ^a^	452.84 ± 21.68 ^a^
**S3**	865.39 ± 44.62 ^a,b^	1106.30 ± 21.36 ^e^	1073.35 ± 37.11 ^d^
**S4**	987.57 ± 14,66 ^a,b,c^	1030.92 ± 10.28 ^e^	882.42 ± 20.72 ^c^
**S6**	831.32 ± 78.12 ^a,b^	815.63 ± 47.03 ^d^	683.97 ± 112.19 ^b^
**S7**	845.94 ± 66.62 ^a,b^	664.60 ± 20.43 ^c^	601.92 ± 7.36 ^b^
**I1**	1079.94 ± 45.08 ^c,d^	442.33 ± 23.39 ^a^	645.77 ± 26.70 ^b^
**C7**	1021.60 ± 93.76 ^b,c^	570.56 ± 18 ^b^	436.43 ± 28.88 ^a^
**C8**	1231.60 ± 118.03 ^d^	624.76 ± 44.78 ^b,c^	651.49 ± 46.72 ^b^

Different letters in the same column mean statistically significant differences (*p* ≤ 0.05) from control samples.

**Table 5:** Antioxidant activity (μM Trolox equivalent) of waste broth with 2% glucose fermented by the selected bacteria at 37 °C for 24, 48, and 72 h.

Sample	24 h	48 h	72 h
**Control**	1160.11 ± 95.75 ^a^	1968.92 ± 104.04 ^b^	1629.99 ± 63.39 ^bc^
**S3**	2559.33 ± 271.05 ^c^	4797.41 ± 407.22 ^e^	2370.39 ± 248.17 ^d^
**S4**	1474.13 ± 5.29 ^a,b^	3726.24 ± 64.46 ^d^	1355.54 ± 44.41 ^b^
**S6**	1218.92 ± 47.74 ^a^	1189.09 ± 13.98 ^a^	1085.19 ± 22.04 ^a,b^
**S7**	1243.28 ± 74.25 ^a^	3058.00 ± 71.93 ^c^	1223.49 ± 75.49 ^a,b^
**I1**	2228.97 ± 129.09 ^c^	2256.52 ± 54.28 ^b^	2130.25 ± 59.39 ^c,d^
**C7**	1362.59 ± 18.57 ^a^	1140.01 ± 39.86 ^a^	603.14 ± 39.86 ^a^
**C8**	1714.81 ± 60.56 ^b^	2016.59 ± 147.17 ^b^	6502.60 ± 638.78 ^e^

Different letters in the same column mean statistically significant differences (*p* ≤ 0.05).
